# Supplementary material for: Identification of Epigenetic Biomarkers of Adolescent Idiopathic Scoliosis Progression: A Workflow to Assess Local Gene Expression
Source: Int J Mol Sci. 2024 May 14;25(10):5329. doi: 10.3390/ijms25105329 (PMC11120692; doi:10.3390/ijms25105329)
Supplement: Supplementary file 1 [file ijms-25-05329-s001.zip › ijms-2951903-supplementary.pdf]

International Journal of *Molecular Sciences*

MDPI

Supplementary Material

Identification of Epigenetic Biomarkers of Adolescent Idiopathic Scoliosis Progression: A Workflow to Assess Local Gene Expression

Simona Neri [1,*], Elisa Assirelli [1], Marco Manzetti [2,3], Giovanni Viroli [2,3], Marco Ialuna [2], Matteo Traversari [2], Jacopo Ciaffi [1], Francesco Ursini [1,3], Cesare Faldini [2,3] and Alberto Ruffilli [2,3]

[1] Medicine and Rheumatology Unit, IRCCS Istituto Ortopedico Rizzoli, 40136 Bologna, Italy; elisa.assirelli@ior.it (E.A.); jacopo.ciaffi@ior.it (J.C.); francesco.ursini@ior.it (F.U.)
[2] 1st Orthopaedic and Traumatologic Clinic, IRCCS Istituto Ortopedico Rizzoli, 40136 Bologna, Italy; marco.manzetti@ior.it (M.M.); giovanni.viroli@ior.it (G.V.); marco.ialuna@ior.it (M.I.); matteo.traversari@ior.it (M.T.); cesare.faldini@ior.it (C.F.); alberto.ruffilli@ior.it (A.R.)
[3] Department of Biomedical and Neuromotor Sciences (DIBINEM), Alma Mater Studiorum University of Bologna, 40126 Bologna, Italy
* Correspondence: simona.neri@ior.it

Table S1. Array based gene expression analysis. Expression of 32 putative housekeeping genes was investigated in bone, muscle, and ligament tissues from 3 AIS and 1 control donors. Cycle threshold (Ct) values are indicated. B AIS= adolescent idiopathic scoliosis spinal facet; M AIS= adolescent idiopathic scoliosis paravertebral muscle; L AIS= adolescent idiopathic scoliosis ligament; B CTR= control spinal facet; M CTR= control paravertebral muscle; L CTR= control ligament.

	B AIS1	B AIS2	B AIS3	M AIS1	M AIS2	M AIS3	L AIS1	L AIS2	L AIS3	B CTR	M CTR	L CTR
**18S**	16,297	22,326	17,39	9,187	18,699	9,277	15,953	10,854	14,923	22,572	9,234	15,060
**GAPDH**	26,121	33,322	30,375	18,364	28,138	18,960	28,121	23,290	28,097	36,963	21,220	26,866
**HPRT1**	29,503	35,592	34,695	27,294	32,308	27,291	32,119	27,898	32,296	>40	27,959	31,815
**GUSB**	28,539	34,450	34,947	28,180	31,217	27,590	>40	>40	>40	>40	>40	>40
**ACTB**	35,188	>40	>40	24,617	31,653	25,384	29,105	23,609	28,987	>40	26,440	29,264
**B2M**	24,783	30,856	29,896	21,259	24,137	21,064	24,798	21,477	26,407	34,483	23,325	26,121
**HMBS**	28,792	34,219	34,891	28,902	34,522	28,737	34,947	32,105	34,030	>40	29,160	33,904
**IPO8**	30,720	36,022	35,526	27,187	31,470	27,221	31,485	26,970	30,066	>40	26,935	30,133
**PGK1**	25,870	32,838	32,656	22,384	29,461	23,558	28,323	25,142	28,924	39,542	24,724	28,327
**RPLP0**	25,840	31,668	27,000	22,152	27,250	22,301	27,640	22,940	27,063	>40	23,117	27,445
**TBP**	33,936	>40	38,519	29,138	38,233	28,830	33,424	28,980	32,507	>40	29,423	33,057
**TFRC**	28,201	32,965	35,707	26,129	31,991	25,891	31,975	29,127	33,510	>40	25,457	31,926
**UBC**	25,884	31,862	32,225	20,639	27,414	21,388	25,971	21,260	24,930	35,464	22,100	26,177
**YWHAZ**	27,322	34,969	34,780	29,835	32,918	31,073	32,524	30,238	34,581	>40	33,381	33,974
**PPIA**	24,299	26,932	28,295	24,419	27,730	24,408	27,849	23,745	26,989	28,284	24,953	25,623
**POLR2A**	29,273	32,851	33,598	27,199	29,952	26,988	30,003	27,560	28,476	38,615	28,144	31,120
**ACTN2**	30,043	35,487	32,997	26,183	29,939	26,357	29,781	25,852	29,633	>40	26,860	29,198
**ATP1A2**	31,215	38,747	35,494	24,987	32,906	25,880	28,056	27,311	29,980	>40	30,954	32,417
**BAG4**	28,937	34,111	34,111	25,931	29,746	25,317	30,106	25,834	29,514	38,131	26,126	30,035

**C1orf61**	31,809	37,187	36,142	26,651	34,989	29,342	33,966	28,911	34,050	>40	31,175	35,460
**CD58**	29,434	35,028	34,307	25,116	29,989	25,351	29,149	25,728	28,013	>40	26,429	29,477
**CENPA**	31,241	37,355	35,414	26,689	31,571	26,867	31,909	28,124	31,714	>40	27,556	31,217
**DCT**	28,570	35,601	34,503	27,102	32,766	27,340	32,893	27,833	31,024	>40	28,205	31,738
**GABRD**	29,170	32,848	31,413	27,973	29,840	27,911	30,158	28,116	28,358	34,866	28,544	30,359
**JAK3**	33,234	37,411	35,488	26,879	31,744	27,181	30,070	26,278	29,925	>40	28,553	31,107
**PLA2G2A**	27,505	33,210	33,980	25,053	29,004	25,506	28,846	25,524	29,016	>40	27,192	29,955
**RXFP3**	19,398	22,442	20,440	16,431	21,632	16,186	21,974	19,945	20,581	21,073	17,641	21,505
**SFPQ**	31,041	37,070	34,982	26,376	32,207	26,763	32,976	28,338	30,531	>40	27,315	31,521
**SHH**	29,260	35,316	32,850	27,844	32,563	27,990	33,325	28,839	32,831	>40	28,893	31,842
**VCAM1**	24,922	28,433	27,135	21,219	27,804	21,848	>40	>40	>40	>40	>40	>40
**WNT1**	28,002	35,534	29,863	24,685	32,589	25,385	29,582	25,912	30,298	>40	25,449	29,334
**WNT2**	23,228	25,588	25,065	22,981	27,311	22,910	25,699	23,359	26,698	26,053	23,245	25,519
